# Opportunities for meaningful inclusion: experience of individuals with intellectual and developmental disabilities with research

**DOI:** 10.3389/fped.2025.1478000

**Published:** 2025-01-29

**Authors:** Ally Dudley, Tai Baker, Canyon Hardesty, Eric J. Moody

**Affiliations:** The Wyoming Institute for Disabilities, University of Wyoming, Laramie, WY, United States

**Keywords:** intellectual and/or developmental disabilities (IDD), journey mapping, research participation, co-researchers, engaged research

## Abstract

**Background:**

Individuals with intellectual and developmental disabilities (IDD) face numerous health disparities, particularly in rural communities. However, they are rarely included in the research process to address these challenges as co-researchers. Little is known about the experience of how individuals with disabilities participate as co-researchers, or the barriers they face.

**Objective:**

The current study explores the experiences of individuals with IDD as co-researchers through discussions with individuals with IDD themselves, those who support them, and disability researchers.

**Method:**

Data were collected through focus groups with individuals with IDD, individuals who support those with IDD, and disability researchers. Each group was asked about their journey through the research process, from beginning to end. Data were analyzed thematically by two independent coders.

**Results:**

While all groups viewed the inclusion of individuals with disabilities as co-researchers as valuable, many barriers still prevented this population from fully participating in the research process. Individuals with IDD viewed research positively, especially when the topics were personally relevant. However, many thought research was intimidating and wanted additional support. Support providers expressed that the people they support have lots to contribute to research and felt empowered when participating. Disability researchers discussed many barriers to include individuals with IDD as co-researchers, including limited time, resources, and inflexibility of research processes. Researchers felt they could use more experience working with individuals with disabilities as co-researchers to integrate these individuals into all aspects of the process.

**Discussion:**

There is broad interest in including those with IDD as co-research, but many barriers remain. Full inclusion can be supported by developing a welcoming and accessible environment. Researchers may need institutional support and training to pursue inclusive IDD research. Asking individuals with IDD for their expertise, develop topics of research that those with IDD can relate to, and involving support providers may be helpful. Developing innovative strategies to support inclusion is needed from all groups.

## Introduction

Individuals with intellectual and/or developmental disabilities (IDD) face persistent healthcare disparities. For instance, those with IDD have poorer overall health outcomes, such as an increased likelihood for obesity and physical inactivity, and a decreased likelihood to receive screenings for cervical, breast, and prostate cancer (1). This population also has increased use of emergency services related to social determinants of health (2). Those with IDD also have higher rates of chronic physical and mental health conditions, emergency department use and 30-day readmission rates (3). Adults with IDD also have significant gaps in their sexual health knowledge (4), have higher rates of interpersonal violence (5), and have higher rates of adverse childhood events and associated long-term negative health consequences (6). Overall, those with IDD struggle with numerous health issues related to access, knowledge, communication and quality (7).

Unfortunately, these negative health outcomes are exacerbated for those with IDD who live in rural settings (8). This is not unexpected given that those in rural and frontier communities experience lower life expectancy and poorer overall health status (9). Rural populations have reliably lower County Health Rankings in areas such as behavioral health, morbidity, and access to clinical care (10). County Health Rankings measure the population health of counties based on interconnected factors related to the policies and procedures of local, state, and federal governments with health factors and health outcomes such as length and quality of life (11). This is strongly influenced by individual and environmental characteristics, including higher rates of risky health behaviors, limited financial resources, socioeconomic differences, limited access to health care, poor health care quality, limitations in infrastructure, insurance deficiencies and a weak public health policy environment (12, 13). These disparities affect all rural patient demographic groups (14) and disability status only compounds these challenges. Therefore, rural residents with IDD are far more likely to face significant health disparities.

Health research is typically a critical step in determining how to remediate highly prevalent health disparities, such as those listed above. Indeed, this has been the cornerstone of public health research for over 100 years. More recently, there is a growing interest in how to bridge the gap between researchers and individuals in impacted communities. New models of community engaged research (15) provide a path to conduct research *with* the community, rather than *on* the community and may produce more locally relevant outcomes. Community-based participatory research (CBPR) is a type of engaged research that prioritizes the inclusion of all stakeholders in all phases of the research process. CBPR research is a collaborative process that includes community members as *co-researchers* in the development of the research questions, methods, data collection, data analysis, dissemination, and determining how the findings will be used to benefit the community (16). A growing body of evidence suggests that programs that draw upon the experience of community members to identify health priorities that matter most to them are more likely to build healthcare capacity and quality in rural communities (9) and for those with IDD (17). The use of CBPR is of particular interest with IDD and rural communities because it is more effective in developing solutions that fit unique local circumstances and, therefore, maybe more likely to lead to sustainable positive change. This is particularly important for extremely rural and underserved communities that have very little capacity to try new programs. Therefore, it is it is important to enhance collaboration among all stakeholders that align with the common interests of the community and professionals (18) and tailor this method of research to better conduct research with individuals with IDD and those in rural communities.

Unfortunately, little is known about the experience of those with IDD in health research, nor how researchers who conduct health research related to IDD include those with IDD in their work. The research needed to remediate these disparities has traditionally excluded those with IDD from the process of research (17, 19). In fact, there has been a long-standing tension between researchers and individuals with IDD, further complicating participation in research by individuals with disabilities (20). Further, given the vastly different healthcare challenges in rural areas as compared to urban areas, solutions that are developed for urban centers are not likely to be effective in rural communities (21). This includes economic factors, cultural and social differences, lack of public health education, political factors and the sheer isolation of living in remote areas all conspire to impede rural Americans in their struggle to lead healthy lives (14). Therefore, solutions that are designed to meet the unique needs of those with IDD living in rural America are desperately needed.

To encourage inclusion of those with IDD as co-researchers, it is essential to understand the experiences individuals with IDD have already had in research, as well as what motivates these individuals to help with research and what barriers they experience. One process that has been used to understand the experiences of individuals in various settings is called journey mapping. Journey mapping is a process commonly employed by healthcare administrators in order to understand care management from the patient's perspective (22). Instead of viewing the patient experience through the healthcare system perspective that may prioritize specific events that are salient to the healthcare system (e.g., patient seeks help, care provider makes diagnosis, patient receives treatment), journey mapping focuses on every activity or event between the patient and their healthcare system that shapes the overall experience (23, 24). For example, journey mapping includes prescription or appointment reminders as well as direct contact with providers in order to identify any gaps in the patient's experience (23). Therefore, the journey mapping process focuses on the whole system, and prioritizes the respondent's point of view.

Journey mapping can be tailored to any population or situation by exploring key experiences, including successes and challenges, that populations face throughout their participation in specific scenarios. For example, in CBPR, co-researchers are expected to contribute to all steps of the research project, including formulation research questions, data collection, data analysis, and dissemination of project results. Utilizing journey mapping as a research tool would require researchers to ask participants about their experiences with all aspects of the research process to further understand barriers to full participation of co-researchers. This tool can then be further modified to focus on the experiences of specific populations, such as the experiences that individuals with IDD have had contributing to research. Further, journey mapping becomes particularly powerful when the experiences of multiple stakeholder groups are considered, compared and contrasted. This allows for a better understanding of the entire social system at a functional level, and uncovers novel areas for intervention.

Therefore, the current study uses journey mapping, adapted to the process of inclusive IDD research to explore the experiences of (1) individuals with IDD, (2) their support providers and (3) disability researchers in the inclusion of individuals with IDD in the research process. Discussions with these three groups will help to identify previous experiences with research, as well as any successes and challenges in integrating individuals with IDD as co-researchers. Further, the current study will provide guidance to multiple stakeholder groups on how to address barriers to the inclusive IDD research. The involvement of individuals with disabilities as co-researchers will allow for more inclusive research, and hopefully lead to valuable research outcomes for individuals with IDD.

## Method

### Participants

Participants fell into three groups: (1) individuals with IDD, (2) individuals who support someone with an IDD (e.g., family members, case managers, direct support providers, etc.), and (3) researchers who engage in research related to disability. Participants were required to be 18 years of age or older. Anyone who identified as someone with an IDD was included in the IDD group. Proof of a formal diagnosis was not required. Individuals with IDD who were their own guardian or had a guardian were permitted to participate. All participants received $50 for their time, given as a gift card.

A total of 9 individuals with IDD, 5 researchers, and 4 people who support someone with an IDD participated in the focus groups. All participants worked and/or resided in Wyoming. Of the participants from the supports group, 1 participant was a family member, 1 participant worked with individuals with IDD, and 2 participants had a dual role of family member and someone who worked with individuals with IDD. All 5 researchers conducted research related to disabilities. Groups were recruited in two ways. The IDD group and supports group were recruited at the Wyoming Developmental Disabilities Conference held in June 2023. All attendees of the conference were invited to attend a pre-conference workshop that explained what research was and the goals of the current research project. Interested participants completed the eligibility and consent process during this workshop. Researchers were recruited through outreach to the University of Wyoming faculty email list, the University of Wyoming College of Health Sciences faculty email list, and the Equality State Research Network (ESRN). Focus groups for researchers were held via Zoom.

This research was determined to be a low-risk study; however, because of potential participants with IDD are part of a protected class, institutional review board (IRB) regulations stipulated that individuals with IDD must demonstrate understanding of the purpose of the research, their rights, potential risks and benefits prior to participating. The individual collecting consent would ensure understanding by asking the individual to repeat back key information about the study, including, but not limited to the purpose of the study, their right to choose to participate or not, that they could stop participating at any time, and that they could choose not to answer any of the questions. This understanding check was conducted prior to consent, and only one individual was unable to complete the comprehension check. If the participant had a legal guardian, a researcher contacted the guardian via email or phone call to obtain approval to discuss the project with the participant prior to administration of the capacity assessment. If the participant demonstrated a capacity to consent and was their own guardian, a written consent form was presented to the participant. If the participant had a legal guardian, the guardian received a consent form, and the participant received an assent form.

### Procedure

Three focus groups with self-advocates and care providers were conducted at the 2023 Wyoming Developmental Disabilities Conference. Researchers from the Wyoming Institute for Disabilities (WIND) held two focus groups with individuals with IDD and one focus group with individuals who support someone with IDD. Two focus groups with IDD researchers were held via Zoom. Focus groups lasted around 60 min. Participants were asked what experiences they, or the individuals they supported, had with IDD research. Participants were encouraged to share successes, difficulties, barriers, and thoughts about including individuals with disabilities in the research process, including creating research questions, collecting and analyzing data, and disseminating results. Interviewers were all staff and faculty of the WIND who had experience working with individuals with IDD.

### Measures

Data were collected using a structured interview guide. The guide was built around the core principles of CBPR (16) to capture experiences with each of the phases of the research process. Questions were specifically tailored for IDD participants, their support providers, and researchers resulting in eight open-ended questions, with follow-up questions (see Supplemental Materials). These questions focused on general experience with research as well as specific questions about developing research questions, data collection, data analysis, and dissemination. Follow-up probes were used to gather more detailed information and to refocus the conversation, as needed. Focus group questions were developed based on the Levy method of journey mapping by determining touchpoints, timeline, external influences, internal influences, and barriers to participation in research (22). To further tailor this tool, researchers discussed experiences relevant to the participant groups in this study, rather than focusing on healthcare systems and their patients as the tool was originally developed for.

### Data analysis

Following the collection of data, transcripts were generated using an online AI-based transcription platform. Prior to analysis, a research associate listened to each interview along with the transcript to correct errors. Transcripts were then analyzed using a thematic analysis approach to determine reoccurring themes across participants and groups based on the shared experiences of participants (25). A thematic analysis approach was chosen to gain in-depth insight into the experiences of individuals with IDD, their supports, and IDD researchers, and explore the similarities and differences of each participant and group. Furthermore, thematic analysis aligned with the goals of the current study, which were to gather the experiences of each group in their own words. Collecting and analyzing data that corresponded directly to the thoughts and experiences of each group allowed for an authentic representation of the journey these participants have had in research.

Several steps were taken to ensure credibility in the findings. The research team comprised of an interdisciplinary team with two faculty members and two staff members. Faculty included CH (public health and qualitative methods) and EM (social psychology and epidemiology). Staff were AD (clinical psychology) and TB (public health and administration). One of the team members identifies as a family member of individuals with IDD. This diversity of perspectives provided a unique lens to this study data.

We also made attempts to enhance the credibility of findings by taking a team approach to data analysis. Two independent coders (AD, TB) listened to each recording before comparing common themes. Coding was completed manually by each coder. To begin this process, each coder read the transcripts from each focus group to become familiar with the data in general. In the initial coding phase, coders used an inductive coding (26) approach called line by line coding to ensure all data was thoroughly analyzed. As coders read through each comment made by participants, they would code lines with descriptors that synthesized the general meaning of the comments. When this process was complete, coders organized their codes into common themes. Following this process, the coders met to compare the themes that emerged and read through transcripts while marking common themes discussed by each group. When coders disagreed on themes, they discussed what evidence was placed under that theme to determine if there was a match to another theme that was categorically similar. Additionally, if one coder split themes into two categories, while the other coder combined those themes, they discussed whether the themes were distinct and came to a consensus on whether to split or combine themes. If a theme arose that only one coder recognized, coders would review the evidence behind that theme and decide together if it should be included in the final list of themes, integrated into another theme, or disregarded. The coders then discussed emerging themes that were in common and developed a final list of themes. These themes were then discussed amongst the entire author group to develop consensus. Several themes were identified for each of the groups, which are outlined below.

## Results

### Individuals with IDD

Four main themes emerged from the focus groups held with individuals with IDD: (1) Positive View of Research, (2) Personal Topics, (3) Research is Intimidating and Inaccessible, and (4) Increasing Comfort in Research. While participants had an overall positive view of research, most had very little experience with any form of research, and those that did, only as a research participant (i.e., as a subject). None had experience as a co-researcher or providing any supportive role to a research team.

#### Positive view of research

Overall, participants indicated that they had a positive view of research. As noted above, none of the participants had ever served as a co-researcher, but many had been research participants. This mostly included answering questionnaires and interviews. However, most participants with IDD spoke of valuing the ability to be a part of something important and helping to solve problems that are important to them. When talking about helping to form research questions, one participant expressed interest in being involved in creating research questions, noting the potential for unique contributions as a result of having an IDD: “*My brain is different, okay my brain. And my brain is different.*” This comment was made in the context of being able to offer a unique perspective as a result of these differences.

Many comments suggested a deep appreciation of participating in research in any way. Several comments focused on how it allows them to use their voices in ways that they usually were not asked to. “*I think it kind of forces you to think creatively about different ways to solve the issue or find solutions to the theme you're researching. Because maybe the typical route to it isn't always the route that works*.” This further suggests an awareness that the unique contributions of people with IDD can help enhance research.

When participants were asked if they were interested in participating in similar research in the future, most participants enthusiastically said they would. When the group was asked if they liked helping with research, one participant stated, “*Yeah. I like it, I like it here. People helped me out. To talk about research. I liked it here*.” Another participant expanded on this by saying,

For me what I like about it is, I know that it helps to educate people and make sure that they are getting the services that they need, not only for myself, but it helps educate … people about disabilities, I really feel like it’s important to educate people on the needs and disabilities and I’ve always thought that since I have a voice, why not be a voice for other people that don’t have a voice. And really get that education out there. And I felt like surveys are a great way to advance Disability Services. And anytime I could be a part of that. I feel like it’s very important. So, I take it very seriously. And I automatically, whenever I can, I automatically always participate in surveys whenever possible.

#### Personal topics

When asked about what topics they hoped to see in research, and what research questions they would like to develop, participants focused on topics that had a personal connection to their lives. Perhaps unsurprisingly, there were interests that extended well beyond healthcare research. For instance, participants discussed health and wellbeing, relationships, jobs, community accessibility, and disability policy. Each of these topics was discussed in relation to personal experiences in the participant's own life. For example, some participants were interested in research regarding social security benefits and marriage, while others were interested in research related to jobs for individuals with IDD.

Each of these topics had a personal impact on the participant that made the comment, and respondents usually could provide clear explanations of the personal value to their life. For instance, several participants had specific interest in health, with one noting an interest in understanding diabetes better: “*Like, figure a way, like, to be healthy*”. Non-health related topics of interest often related to social structures, healthcare systems or policies that had significant impact on their lives. One respondent noted a strong interest in potential problems related to an upcoming marriage and how it could impact his social security and other benefits: “*… whenever we get married, I'm going to be losing all of my SSI benefits … And we're trying to, and I'm trying to figure out why that is or what the reason is behind it.”* Several other comments focused on health care access, especially through Medicaid. The common theme among these topics is that they are meaningful to the participants with IDD and the results could have an impact on their lives.

#### Research is intimidating and inaccessible

One of the major barriers preventing those with IDD from participating in research was a concern that research is too intimidating and hard to understand. This included several unique facets, but focused predominantly on not understanding the research process, and inaccessibility of materials. For example, most participants indicated that they would not know what to do if they were asked to help with research. This seemed to relate to a general lack of knowledge about the research process. However, most suggested that they would be willing to learn. For instance, several participants stated that they would need to start with smaller tasks to help build their confidence before they were able to feel comfortable helping with a research team. When talking about getting involved with data collection and interviewing research participants, one participant talked about starting with smaller, less intimidating tasks:

I’d start with people I know first and then slowly work my way up to people I don’t know. Because I’m one of those that like feed off of… encouragement. Like, if I know that…I could get somebody I know to fill out a survey and I’m like, okay, I kind of got this.

Other participants talked about inaccessibility of materials. Several mentioned how research can be hard to understand, especially when words are used that they do not understand, or instructions are not repeated. During a discussion about not knowing what research means, one participant stated, “*Yeah. Especially when you use big fancy words*”. Another participant agreed that understanding what was being discussed could be difficult, and they would need things repeated to them in order to understand, “*…please repeat it. Again, and hard to understand. hard, hard to understand. Like, like, you know what that means?”*.

Difficulty with traveling to where the research is conducted was also commonly discussed. One participant noted “It’s kinda..Well research..What’s this? It’s really hard for me, it’s hard for me because I’m doing this on my own. I left [Wyoming town] last night and then come here [Wyoming Town – about 150 miles away].” This respondent noted that this amount of travel was particularly difficult and made it hard to participate in research, which was echoed by others.

These concerns also led to participants also worried about being rejected by researcher and fear of social situations related to research such talking to people they did not know when collecting data. While discussing their fears of helping with data collection, a participant said, “*Especially walking up to a complete stranger. It's like, this is a con. Yeah”*. Overall, this suggests that research is both intimidating and inaccessible at times for individuals with IDD, but individuals are willing to learn and grow their skills.

#### Increasing comfort in research

Despite their concerns about joining a research team, several participants had ideas of how they could be more successful as a co-researcher. When discussing how to get involved with research indicated that they would feel more comfortable if they were able to participate with someone who would provide support to support increased engagement in research. This support related to several technical components of participation such as scheduling, transportation, and use of technology to complete surveys. Respondents noted that they thought a support provider could help them manage the research process better and ease any discomfort individuals with disabilities may have. Interestingly, this was not exclusively a professional support provider (e.g., DPS), but could include people from a variety of formal or informal roles. For instance, when participants were asked who they would like to be present to help with research, participants had many ideas including, “*Maybe an older, an older person in your life*”, “*a mentor, or like a coach, or a teacher*”, “*family member, a case manager*”, “*provider*”, and “*friends that you talk to*”.

Indeed, many respondents pointed out that building relationships with peers with similar research interests may help them engage in the research process. When talking about the conference where the focus groups were held and avenues where individuals with IDD could participate in research, participants generally agreed noting:

Maybe like so if you all wanted to help us actually collect data, this might be a place where we maybe we train you up beforehand, and maybe talk with job coaches, and case managers and say, “We want you guys to help with this.” And we can even make it a bit of a your job, right? pay a little bit of money. And you could help us collect data.

Generally, many participants thought that working in a group of people with similar experiences and interests would increase their comfort to talk about the research that they are passionate about. When discussing how to get research started a participant stated:

And then you guys can all. You guys, all of us can just all like group together and not essentially peer pressure. But peer pressure these people into helping us answer the questions and doing the research that we [want].

### Support providers for individuals with IDD

Like the IDD group, most support provider participants reported their clients and/or family members had participated in some sort of research in the past but had not been involved as a co-researcher. These respondents generally regarded research as a positive experience for their clients and loved-ones. Five themes emerged from the support providers: Enthusiasm to Participate, Empowerment, Inclusion, Safe Environment, and Translatable Skills.

#### Enthusiasm to participate

A very strong theme emerged from this group indicating a belief that their clients would be interested in participating as co-researchers. Overall, there was high enthusiasm for participation and each person stated that if the people they support were given the opportunity to help with research, they would take it. One participant stated, “*Like some of my participants, they would be here in a second telling you how much they love you. And they cannot wait and give you the answers that you're looking for*”. Another participant mirrored these thoughts when it came to a family member, “*…once she gets involved, she's more than ready to give information about what her life or her experience has been*”. A third participant agreed that some of her clients would be interested in participating in research:

I can think of three or four [clients] right now that they would love that. They would love to be able to say, you know, these are the types of conversations that I want to have with somebody and so maybe somebody else would too.

This group made it clear that the people they support want to be heard if people are willing to ask them about their experiences.

#### Empowerment

Participants from the provider group discussed that participating in research has allowed the people they support to feel empowered. These respondents noted that when individuals with disabilities are asked to participate in research, they are excited to talk about their experiences and have feel like they have some control over the research that they are helping with. A family member of an individual with IDD said, “*I think it's a sense of inclusion. And accomplishment that someone's listening to me*.” Additionally, participants noted that having an opportunity to talk about their experiences in a group setting is empowering because there are often like-minded individuals who have had similar experiences listening. One support provider stated:

I mean, at least in what I’ve seen from my participants, even if we … break down … the survey into like a smaller degree, and not just like, in this sort of capacity. I feel like there’s always like a sense of empowerment, because typically you’re in a group setting, there is the capacity of being around people who can relate.

Further, participants commented that using research as a platform to advocate for oneself and for others with disabilities is very impactful for the people they support. They like that their contribution to research could help other people with IDD. One participant stated, “*that's where I think my clients would be like, yes, we need this information so that other people, you know them included, have that benefit*”. The empowerment from participating in research makes it more likely for individuals to seek out research, as well as other empowering activities, in the future.

#### Inclusion

Participants in this group expressed that the people they support want to be asked to participate in research and they want to be listened to throughout the whole process. They indicated that this would promote a sense of empowerment and make individuals with IDD feel respected. Several respondents also noted that in previous experiences with medical appointments and research experiences, individuals with IDD were usually not asked questions directly. One participant reflected on this:

I think one thing that happens quite often, and whether it’s an interview or a questionnaire, those people asking the questions, if you are with your child, or your person you’re advocating for, or you’re, if you’re a respite worker, they always tend to ask the adult or the normal physical person instead. And so many times I said, don’t ask me, ask her… And so,… I find that happens quite often in this kind of informational part where they start asking questions, they need to ask the individual and see if they are capable or not. But they jumped to conclusions.

Additionally, participants in this group emphasized a need for communication throughout each research step, including improved communication during data collection and dissemination of results. One participated noted that in a research study, they stopped receiving communication from the researchers, “*Yeah, you expect a kind of a follow up or reason, or maybe we've terminated this information or research that just dropped with no reason or rhyme*”. Many participants noted that the people they support never received information about the results of the studies they participated in, leaving them confused about the outcome of the study and how it impacted individuals with disabilities. One participant stated:

Yeah, you know, where are the results? Yeah, we did this stuff. And I never got anything. You know, my daughter did this whole thing. And it was a couple of years. I have no idea what their results were… How did it help them? What did they get out of it? I have no clue. And I have no way to get that information. So, I know that, you know, my daughter doesn’t have that information. My clients don’t have that information.

#### Safe environment

When discussing the ideal circumstances for individuals with disabilities to participate in research, many support providers discussed the need for a safe environment. This group noted that individuals with disabilities need to know that they will not be judged for what they say and that they are in an environment where they are allowed to freely express themselves. One example of a safe environment was the conference where the focus groups were held. When asked if the conference felt like a safe environment for individuals to express themselves, one support person said:

100% Because they are with their like-minded peers, they are with new peers. There’s new things, and then you’ve got this amazing speaker who’s just making you feel all kinds of happiness? Absolutely. Absolutely. Yes. It’s more an open environment.

Respondents noted that this type of open environment develops a sense of comfort for individuals with disabilities and creates an opportunity in which they can formulate their own stories to share with researchers and their peers. Participants stated that the people they support are often left out of conversations or feel they are in environments that are too restrictive for them to share their thoughts. When discussing another speaker at the conference that had talked about his experiences not being included due to his disability, a family member shared:

Yeah, the one individual today, he had such a powerful message. But if you looked at him, you would think he was angry. But he wasn’t. He was so happy to say what he said. And it’s like I just applauded him for. And I don’t know, if you would put him in a more open environment, people would have been maybe offended, by the way he approached that, just the way he looked. And it doesn’t matter how he looked. His message was very important. Yeah, so. But it was good that he had the courage to say what he wanted to say, and everybody listened.

#### Translatable skills

Most participants from this group reported that the people they support did not have any formal experience in helping with any aspects of the research process. However, many participants discussed skills and experiences that they thought would be beneficial in supporting research projects. Specifically, participants discussed a range of experiences and skills related to critical thinking, resourcefulness, and problem-solving. For instance, when asked if the people they supported had ever interviewed participants, one support person stated the people they supported participated in the hiring process at their group home and asked potential staff questions during the interview process, “*It's just interviews that they will give somebody who we'll hire. Alright, so they were doing the interviewing on if they're going to hire them or not.*” Additionally, when participants were asked if the individuals they supported had ever assisted with planning a study, one participant stated that their daughter had organized a birthday party.

…when my daughter was going through transition classes, and you know, past 18, but not 21 yet. You know, they would sit down, and the group would have a discussion on a, okay, well such and such birthdays coming up. So, we’re gonna have, we’re gonna do like a lunch. We’re gonna go to the park and have a picnic. And so, then she would automatically take the lead and get everybody’s information. And then she would compile that and talk about it. And, okay, well, this is what most people want. And so, this is what we’re going to do. And now we need to make a budget, so we know get those things. And then we have to put those things together. So, she would direct. And I think she would take that information and kind of figure out what was going to work in the process. So yeah, it’s the same. Like, it’s the same process.

Other examples of experiences with translatable skills included participating in science fairs in school, participating in speech and debate clubs, propagating plants, and counting the number of new animals added to a family's goat herd. In general, this group thought that there were many skills that people with IDD can use to support research projects.

### Disability researchers

Like the previous two groups, the group of researchers noted that those with disabilities rarely ever participate in research as co-researchers. This group noted several obstacles that discouraged the inclusion of individuals with IDD in research. In discussions about these obstacles, four prominent themes emerged: Structural Barriers, Comfort/Training, Advisory Boards, and Disconnect Between Academic Processes and What Individuals with IDD Need.

#### Structural barriers

The most prominent theme among disability researchers were the structural barriers that they faced in including individuals with disabilities in the research process. The most common barriers discussed were resources (i.e., money, childcare, translators) and time. In relation to resources, researchers discussed how it was difficult to find funding to compensate participants and research assistants. Additionally, finding funding to help co-researchers with disabilities attend conferences was a barrier. One researcher talked about the cost of attending conferences:

So like, like I’m planning on going to [conference] in [month], well that’s in [state]. So it’s like, … actually a requirement of the [grant], which is what I want too. What I’m presenting on, is that I cannot use those funds to pay for a conference. So, I can’t pay for a participant to come with me to go. So, then it’s like, well, how do I get them there? And it’s very costly, too.

During discussions about time, researchers talked about how much of a time commitment CBPR can be. It can be difficult to find individuals, including those with IDD, who are willing to commit a significant amount of time to a project. The time commitment can be intimidating to both researchers and individuals with IDD who may otherwise be interested in helping with research. When talking about time, a researcher commented:

…and if we’re talking about like, participatory research, like that’s a big time investment. It’s not just like, let me interview you and then I’ll give you your incentive. That’s one thing but if we’re talking about, like, being a part of this whole thing, and we really want you invested, Yeah, it’s great when people want to be involved and people volunteer and stuff like that, but I also think people should be compensated for their time.

Related barriers discussed include a lack of flexibility in research plans, such as having inaccessible meeting times for community members, and the inaccessibility of many research tools and spaces. One researcher reflected on the accessibility of the research tools they were using and how they learned from these unexpected accessibility challenges:

I mean, I think it’s always challenging, especially when you’re researchers that don’t presently have a disability. Because there’s always things that we don’t anticipate, that we didn’t plan for, right? And especially in terms of accessibility. Like, for instance, the [name of project] that we did this past year, we brought in these cameras that my colleague had and they were just these little point and shoot cameras, but they were too small for the people and they we didn’t have tripods and some people’s hands shook, you know, and so we just like really learned a lot about, like, okay, we need to go out and we need to get some nail polish to mark certain buttons for them. We need to get tripods we need to get bigger cameras, maybe some iPads, just to make that more accessible.

#### Comfort/training

Although a less common theme, a few researchers discussed a lack of comfort in including individuals with IDD, for both themselves as researchers and for co-researchers with IDD. For some researchers, they were cognizant that they lack the training they need to know how to effectively include individuals with IDD in the research process. These respondents also talked about a lack of experience in conducting research on topics that individuals with IDD may be interested in. For example, one researcher noted a personal lack of technical training to approach questions about larger, census level research questions that some individuals in the community had posed:

…And so it seems to me that the folks in the community have ideas about what needs to be done. And for me, a barrier is that I don’t have any training doing that at all. And some of the ideas that folks have shared with me and these are caregivers and individuals with [disorder] are like census level type of questions, which again is like seemingly so out of my wheelhouse.

This group also discussed a potential lack of training for co-researchers with IDD as well. Specifically, many researchers discussed that many individuals with IDD, although interested in helping, may not have a background in research methods to be effective. Research worried that this could become more complex in later stages of the research process, such as analyzing complex data. While talking about data analysis, one researcher noted:

I think is harder for quantitative analysis, maybe qualitative analysis to come out with a theme of different comments, I think that’d be very valuable. But quantitatively, I think it’d be quite hard to do the stats do the math, that would be harder.

This group expressed concern that researchers must train these new co-researchers, starting with basic research methods. One researcher reflected on a previous research study where individuals with IDD were involved:

One was that I think, kind of a problem that you have, when you’re working with people who don’t engage with research very often is just like, explaining what is a research question. And, and then getting, so, it’s like, basically, you have to teach intro to research, in order to, to involve… involve anyone into in to a research project. And, so that was true of people with intellectual and developmental disabilities, too. So, and that really went back to very basics, we had to ask, like, we had to talk about, like, what is a question? What makes an interesting question? What’s a research question? What makes an interesting research question?

#### Advisory boards

Several researchers reported utilizing advisory boards comprised of individuals with IDD in previous studies but worried about the degree to which this was meaningful or impactful. These advisory boards were largely used as sounding boards in the beginning of studies to ensure that study procedures and the questions that were asked would be appropriate for the target population. In some cases, advisory boards gave some input in the interpretation of data or were given reports of outcomes. One researcher noted:

We brought the results back to the advisory board, and kind of told them what it meant. And, you know, because it’s statistical analysis, yada, yada, yada. And what we were finding and asked them sort of like, well, what do you think about this? How does this make sense to you, like questions like that, but and I think that that process is important, but I don’t know how, how meaningful it is either. Because I don’t think like we did that because it was part of our process. And a step like we were we were dedicated to we… were committed to including the advisory board in every part of the process, and also including them on publications. But I don’t know if we actually included any of that input in our analysis, it was still more just, this is what the statistical analysis says, and this is how the researchers are interpreting it.

Similarly, most researchers reported their advisory boards did not participate in other vital research processes, such as data collection, data analysis, and dissemination. Researchers attributed these deficits in participation to the barriers discussed above, as well as a lack of interest in other parts of the research process. When discussing previous experiences with advisory boards, one researcher stated:

For me with my community advisory council, I did ask if any of them wanted to help as far as interviewing. So, I gave them the option because I didn’t want them to feel obligated to have to. And I certainly didn’t want them to feel uncomfortable doing so. And none of them expressed an interest. So, I didn’t, I didn’t push it, because I was grateful for the assistance in creating the questions.

Further, several researchers commented that they believed they should include individuals with IDD in more aspects of the research process but had not done so due to the numerous barriers noted. One participant stated:

So, I think in that sense, yeah, paying people… maybe actively seeking out research assistants with disabilities that are members of the community that you’re working with? Yeah, yeah. I don’t see why it can’t be done. And it probably should be done more often, I would say too.

Another researcher mirrored those thoughts while reflecting on their own research and how they had not asked people with IDD for their input:

And now I’m wondering, because as like, from my clinical training, I have all these ideas about, you know, what makes the communication environment hard, and what we can do to reduce the load of that communication environment and make it more successful. And now, I just think maybe I should ask [community members]. You know, because I have all of these ideas. I know it’s shocking. I’m really glad we’re having this. You know, I think it’s easy to get lost for me in what we already know. But we haven’t… I haven’t really revisited that. And maybe there’s contexts that these folks are experiencing whether you know, maybe they’re outside a lot on their property, are these contexts that I don’t even know.

#### Disconnect between academic processes and what individuals with disabilities need

One aspect that many researchers commented on was how the typical functioning of academia does not fit well with modified processes that would benefit individuals with IDD. For instance, participants stated that research requires rigorous methodology and changing procedures to be more accessible for co-researchers with IDD may be difficult. One researcher noted, “*it's hard, because it just in the science world we're so strict about how you control everything, is getting, yes, you have to do things by the literature. And it's hard to change those protocols*”. Further, this researcher commented that including individuals with IDD in research may change the way they interact with their university's IRB. They discussed IRB regulations and the extensive training required to follow the rules of research, which may be difficult for co-researchers with disabilities to complete:

Another thing is, IRB board. I think this might change the way I apply for IRB too. Basically, all your concern, you want to ask him, do you want to also be a researcher and then they have to do the training to be a researcher to follow the rules, the confidentiality and minimizing the risks. Just getting a little bit complicated situation here. And then they also need..the full faculty members at [university], we kind of have a liability with [university] that we violate, we can get fined. Right. But then, yeah, people outside not with [university]. I think that’s another thing we..it’s not saying we shouldn’t explore. But I think it would create some other things we have to go through to make sure it can happen.

Further, researchers noted that academia tends to be inaccessible for those outside of a university setting, due to its emphasis on advanced literature and intimidating dissemination processes, such as academic papers and presentations. This group noted that while these processes are commonplace for researchers, individuals with disabilities may be hesitant to engage in research if these barriers are in place and may benefit from different forms of dissemination. One researcher noted how conferences can be intimidating for individuals with IDD, and more informal presentations may be beneficial:

Maybe because of the not so much the attention but just the, the intimidation factor of these are all people that I don’t know and they may be professional and I don’t feel comfortable talking in front of these people that are presenting this information, but things that are more meaningful for them like presenting information to city council because it directly affects them, their community, their friends and neighbors.

## Discussion

Three groups of stakeholders provided their perspective on inclusions of individuals with IDD as co-researchers. In general, all three groups expressed interest in inclusion of those with IDD in the research process. However, all groups identified a variety of barriers noted that make it challenging to conduct community engaged research with those with IDD. Many of these barriers were related to lack of comfort, knowledge and training related to inclusive IDD research. Other barriers were structural and related to the environments in which academic research is conducted.

One commonality between all three groups was an agreement that the inclusion of people with IDD as coresearchers is a worthwhile and important endeavor. For individuals with IDD, research is a platform in which they can express their opinions and contribute to outcomes that matter to them. Participants with IDD felt excited to participate in research where they could talk about their experiences and have an impact on the lives of other individuals with IDD. Similarly, people who support individuals with IDD discussed the inclusion of individuals with IDD as empowering for the individuals they support. When individuals with IDD are invited to advocate for themselves and help shape research, they feel empowered to continue sharing their experiences and engaging with research. Researchers also noted an interest in including individuals with IDD in research, and that it has been useful in the past to include the lived experiences of individuals with IDD when planning projects and interpreting data. Further, multiple researchers expressed that they should be doing more to include individuals with IDD in their research, demonstrating an awareness of the value of lived experience.

While the importance of individuals with IDD as co-researchers was recognized in each group, many discussions turned towards the broad range of barriers that make including individuals with IDD difficult. One such barrier is a lack of comfort related to the inclusion of individuals with IDD. For example, individuals with IDD expressed uncertainty and feelings of intimidation when thinking about participating as a co-researcher. For most individuals with IDD, helping with research is a novel task, and individuals may not know where to start or feel confident enough in their skills to believe that they could provide a meaningful contribution. Similarly, researchers expressed a lack of comfort in including individuals with IDD in their research due to uncertainty about how to effectively include this population in various research tasks. These concerns may demonstrate a need for increased training for both groups to prepare them to effectively participate in inclusive IDD research. This may include developing formal training for self-advocates to better understand what research is, how specific tasks should be conducted and how to contribute to the process. Researchers may also benefit from training on working with those with IDD, how to develop accessible materials, and tailor tasks to the skills of the individuals serving as co-researchers. Additionally, there may be a benefit to promoting more informal meetings between individuals with IDD and researchers. Informal interactions can help build rapport, develop trust and openness, create opportunities to discuss support needs and how to increase comfort with engaged research.

Another notable finding from this study was the potential role of care providers. Individuals with IDD expressed an interest in having trusted individuals, such as family members, staff, mentors or friends, around as they helped with research in the event they needed support or felt uncomfortable. Similarly, individuals from the supports group talked about the importance of creating an open environment that makes individuals with IDD comfortable. This group discussed that when individuals with IDD know they are supported, they are more willing to do things that are out of their normal routine. Support providers also noted that many individuals with IDD have transferable skills that could support research endeavors. This suggests that care providers could be important allies in supporting inclusive IDD research. Given that direct support providers are charged with implementing person-centered plans of care that allow the individual with IDD to engage in desired activities, they may be able to help build engaged research activities into care plans. Other service systems could also be supportive. For instance, Vocational Rehabilitation provides job coaching and other supports for those with IDD to promote competitive employment. These, and other support systems could be particularly important to supporting inclusive research given their roles. This would necessarily include other natural supports, such as family members or guardians who also support loved ones with IDD given that they may have some degree of formal or informal control over some aspects of the individuals’ day-to-day life (e.g., guardianship).

While there was general support for individual measures, such as training or supported employment, that could promote inclusive IDD research, there were also several important structural barriers that could present significant disincentives. Researchers were the group that were most likely to note these challenges, which included a lack of time and resources, limited funding, and institutional limitations such as strict IRB requirements. Structural barriers may be more resistant to change given that they require institutional and/or cultural change. This may discourage researchers, who may have requirements from their institutions to publish papers and acquire grant funding, from attempting CBPR and including individuals with IDD in the research process. This may be especially true for newer researchers who have increased grant and manuscript production demands to secure tenure.

Given the importance of securing funding to advance research, it may be useful for funders to encourage more engaged research, or roles for those with lived experience. While there are some funding mechanisms designed specifically for CBPR, monetary and time costs may still dissuade researchers from pursuing this work if they have institutional demands for research production. Nonetheless, securing significant funding for inclusive and engaged research can help institutions understand the value of this approach and help support researchers that wish to pursue this type of work. Moreover, as more funders recognize the value of engaged research, they may consider building structural features into the award, such as additional time for engagement, CBPR training expectations, and requirements to include co-researchers.

Similarly, the comments related to compliance challenges (e.g., IRB) suggest that research institutions, themselves, may pose significant structural barriers to inclusive IDD research. Certainly, IRBs and research institutions are critical partners in protecting human research subjects and must continue to ensure that research is conducted in ways that do not pose unacceptable risks. However, there may be novel accommodations that IRBs and research offices can make that will allow for more opportunities for co-researchers with IDD, while ensuring the highest level of human subjects protections. For instance, models of co-researcher training that are specifically designed for individuals with IDD while meeting all standard ethical standards [e.g., (27, 28)] can be adopted by IRBs to allow for those with IDD to participate more easily. Trainings such as these contain all necessary learning modules, but do not have the same reliance on inaccessible written language or technical jargon that may create significant barriers to non-professionals. While IRB protocols are vital to protect individuals with disabilities (29), new approaches to training those with IDD may be particularly useful in helping this population understand the research process, their role in engaged research, and help them feel confident in their ability to contribute to the research process.

### Limitations and future research

Although we found clear and consistent themes in this study, there are several limitations that should be mentioned. First, these results were developed from a small sample, from a limited geological range. This was intentional given that the experiences of individuals with IDD living in rural areas are underrepresented in research. While this approach allowed for better understanding an often-marginalized group, it also limits generalizability. Future research may seek out the experiences of individuals with IDD in additional geographic areas to further validate these findings. However, it will be important to further study the experiences of those living in rural communities. Given the health disparities for all rural communities, finding innovative solutions to healthcare problems is paramount. Moreover, inclusion of those with IDD in the process may allow for the development of solutions that are more effective for the local challenges facing rural America.

Additionally, the current research identified a number of barriers that make the inclusion of individuals with IDD challenging. For instance, Wyoming is a rural state with a sparse population, making recruitment difficult. Additionally, many individuals with IDD did not have access to email or phone calls, further complicating the recruitment process. This further limits generalizability. Additional work is needed to identify how to address these barriers; however, this research is instructive. Through the process of engaging with the states Developmental Disabilities network (i.e., Wyoming Governor's Council for Developmental Disabilities) we were able to secure a significant amount of interest and participation in this work, which would have been otherwise difficult, if not impossible. Engagement efforts, such as this, may be the most effective approach to developing more robust samples of individuals with IDD. However, researchers will continue to need to make efforts to meet potential participants in the communities that they live and work and may need to employ multiple strategies to be successful.

Further, future research is needed to address the barriers noted in this research. For instance, a recurring theme discussed in this research is that individuals with IDD and researchers do not feel completely comfortable engaging with each other. It will be important to develop training or other supports to help each group be more secure in working with the other. This may include trainings to make research in this area less intimidating for individuals with IDD, or to help researchers know how to better interact with this group. Or other forms of support that can be delivered by direct support providers could be useful. The role of assistive technologies could also be important, as well as addressing structural barriers that may discourage researchers from pursuing engaged IDD research. Regardless, future research related to developing research skills and increasing vocational efforts in research for individuals with IDD would help encourage more engaged IDD research.

## Conclusion

This study demonstrates that, despite strong interest, there are many barriers that make it difficult for those with IDD to participate as co-researchers. These barriers will need to be addressed to facilitate the participation of individuals with disabilities as co-researchers in IDD research. While individuals with IDD, support providers, and IDD researchers all value inclusive IDD research, additional training and modifications to research processes are needed to promote inclusive IDD research. Although addressing some of these barriers will likely be difficult in the near-term including individuals with IDD will strengthen the research process and the products that are developed through inclusive research. Nonetheless, this research is instructive regarding the nature of challenges that stakeholders face when trying to create inclusive IDD research. This will help focus efforts on addressing these barriers to promote greater inclusion.

## Data Availability

The raw data supporting the conclusions of this article will be made available by the authors, without undue reservation.
