# Supplementary material for: Opportunities for meaningful inclusion: experience of individuals with intellectual and developmental disabilities with research
Source: Front Pediatr. 2025 Jan 29;13:1478000. doi: 10.3389/fped.2025.1478000 (PMC11813933; doi:10.3389/fped.2025.1478000)
Supplement: Supplementary file 1 [file Supplementaryfile1.docx]

**Focus Group Questions: Individuals with IDD**

1. How have you helped with any type of research or project?
2. What was helping with research like for you?
3. What did you like about helping with research?
4. What was hard about helping with research?
5. Have you ever helped choose any of the things that research was going to be about? Have you ever helped make the questions that the research asked about?
   1. If Yes: What was helping do these things like for you?
   2. If No: How do you think you could help with this type of research in the future?
6. Have you ever helped do anything with research like asking people questions about the research or helping them fill out a survey?
   1. If Yes: What was helping do these things like for you?
   2. If No: How do you think you could help with this type of research in the future?
7. Have you ever helped researchers try to understand the information they got from research?
   1. If Yes: What was helping with that like for you?
   2. If No: How do you think you could help with this type of research in the future?
8. Have you ever helped tell people about the research after it was over?
   1. If Yes: What was it like for you to tell people about this research?
   2. If No: How do you think you could help with telling people about this research in the future?

**Focus Group Questions: Researchers**

1. What experiences have you had with research?
2. Have you ever had any experience including individuals with a IDD in the research process?
   1. What was this experience like?
3. What was helpful about including individuals with an IDD in the research process?
4. What was challenging about including individuals with an IDD in the research process?
5. Have you ever included indivudals with an IDD in the creation of research questions?
   1. If Yes: How was this experience for you and the indiviudals with IDD that helped with this process?
   2. If No: How do you think indiviudals with an IDD could help with this process?
6. Have you ever included individuals in the process of conducting research, such as helping with questionnaires or conducting interviews?
   1. If Yes: How was this experience for you and the indiviudals with IDD that helped with this process?
   2. If No: How do you think indiviudals with an IDD could help with this process?
7. Have you ever included individuals with an IDD in data analysis?
   1. If Yes: How was this experience for you and the indiviudals with IDD that helped with this process?
   2. If No: How do you think indiviudals with an IDD could help with this process?
8. Have you ever included individuals with an IDD in the presetation of a research project?
   1. If Yes: How was this experience for you and the indiviudals with IDD that helped with this process?
   2. If No: How do you think indiviudals with an IDD could help with this process?

**Focus Group Questions: Microsystem Supports**

1. What experiences have your clients/family members had with research?
2. What was it like for your clients/family members to participate in research?
3. What did your clients/family members like about participating in research?
4. What was challening about your clients/family members participating in research?
5. Have your clients/family members ever helped with creating research questions? Research questions are the questions that are asked about things we want to learn more about. For example, a research question may be “What do individuals with a disability think about research?”
   1. If Yes: What was this expereince like for them?
   2. If No: How do you think your clients/family members could help with this process?
6. Have your clients/family members ever helped with the process of conducting or running research? This could include tasks such as helping someone fill out a survery or asking questions in an interview.
   1. If Yes: What was this experience like for them?
   2. If No: How do you think your clients/family members could help with this process?
7. Have your clients/family members ever helped with data analysis? Data analysis is done after all the information is collected and researchers want to understand what that information means.
   1. If Yes: What was this experience like for them?
   2. If No: How do you think your clients/family members could help with this process?
8. Have your clients/family members ever helped present the results of a research project?
   1. If Yes: What was this experience like for them?
   2. If No: How do you think your clients could help with presenting research?
